# Intermittent Administration of Parathyroid Hormone Enhances Odonto/Osteogenic Differentiation of Stem Cells from the Apical Papilla via JNK and P38 MAPK Pathways

**DOI:** 10.1155/2020/5128128

**Published:** 2020-02-14

**Authors:** Xiyao Pang, Ying Zhuang, Zehan Li, Shuanglin Jing, Qin Cai, Fengge Zhang, Changao Xue, Jinhua Yu

**Affiliations:** ^1^Department of Stomatology, Nanjing First Hospital, Nanjing Medical University, Nanjing, Jiangsu, China; ^2^Endodontic Department, School of Stomatology, Nanjing Medical University, Nanjing, Jiangsu, China; ^3^Department of Stomatology, Binhai County People's Hospital, Yancheng, Jiangsu, China

## Abstract

**Objective:**

Parathyroid hormone (PTH) is considered to be essential during the tooth development. Stem cells from the apical papilla (SCAPs) are responsible for dentine formation. However, the interaction between PTH and SCAPs remains unclear. This study was aimed at investigating the effects of PTH on odonto/osteogenic differentiation capacity of SCAPs and elucidating the underlying molecular mechanisms. *Materials and Methods*. Here, SCAPs were isolated and identified *in vitro*. Effects of PTH on the proliferation of SCAPs were determined by Cell Counting Kit-8 (CCK-8), flow cytometry (FCM), and EdU. Alkaline phosphatase (ALP) activity, alizarin red staining, Western blot, and RT-PCR were carried out to detect the odonto/osteogenic differentiation of PTH-treated SCAPs as well as the participation of the MAPK signaling pathway.

**Results:**

An ALP activity assay determined that 10^−8^ mol/L PTH was the optimal concentration for the induction of SCAPs with no significant influence on the proliferation of SCAPs as indicated by CCK-8, FCM, and EdU. The expression of odonto/osteogenic markers was significantly upregulated in mRNA levels and protein levels. Moreover, intermittent treatment of PTH also increased phosphorylation of JNK and P38, and the differentiation was suppressed following the inhibition of JNK and P38 MAPK pathways.

**Conclusion:**

PTH can regulate the odonto/osteogenic differentiation of SCAPs via JNK and P38 MAPK pathways.

## 1. Introduction

Dental pulp, a mineralized connective tissue, has the intrinsic ability to form reparative dentine when insulted due to trauma or infection [1, 2]. Clinically, stem cell populations and biomaterials are verified for dental pulp tissue regeneration. As promising dental-derived mesenchymal stem cells (MSCs), stem cells from the apical papilla (SCAPs) have the advantages of no ethical controversy and low immunogenicity. SCAPs reside in the root apex of incompletely developed teeth which can easily be isolated [3]. Besides, it has been demonstrated that SCAPs can differentiate into functional odontoblast-like cells when implanted into the subcutaneous space of mice with scaffold materials [4]. In addition, the role of SCAPs during root formation is observed in many studies [5]. Interestingly, *in vivo* studies showed that root development can be halted when cells were isolated from tooth buds at an early stage [6]. Taken together, the easy accessibility and superior properties established them as an attractive choice for tooth and bone tissue engineering.

Our previous studies have demonstrated that the odonto/osteogenic differentiation capacity of SCAPs can be regulated by many factors including inorganic substance, growth factors, and proinflammatory cytokines [7–9]. However, little is known about whether SCAPs can also respond to hormonal stimulation in an odontoblast-like manner during dentine formation. In this study, PTH is the hormone of our interest. PTH, an endocrine factor, secreted by parathyroid glands plays a pivotal role in the development and differentiation of bones and dental tissues [10]. Previous studies showed that PTH regulates calcium and phosphorus concentrations in the extracellular fluid and blood. Changes in the level of serum calcium result in structural alterations of mineral formation [11]. Interestingly, PTH can induce bone formation and bone resorption in a dose-dependent manner [12]. Specifically, catabolic activity has been assigned to continuous infusion of PTH, as opposed to increased osteoblast effects caused by intermittent PTH injection [13]. Reports have found that PTH activity resides mainly within the 1-34 N-terminal fragment [14]. Synthetic recombinant human PTH 1-34 amino-terminal fragment of parathyroid hormone (PTH1-34) has been shown to participate in the treatment of severe osteoporosis, as it is the only clinically approved drug with osteoanabolic properties [15]. One of its mechanisms is the attenuation of apoptosis of mature osteoblasts. Several studies have shown that 20 *μ*g of PTH1-34 daily accelerates the healing of distal radial fracture in postmenopausal women [16].

Tooth formation is mediated by a series of signaling molecules, receptors, and transcriptional control systems [17]. Mitogen-activated protein kinases (MAPKs) are an essential component in many physiological processes, such as cell proliferation, differentiation, inflammation, transformation, and condensation [18, 19]. The MAPK cascades are composed of three sequentially activated kinase complexes, including p38 MAPK, extracellular signal-regulated kinase (ERK), and c-Jun N-terminal kinase (JNK) [20]. ERK and P38 pathways play an important role in mediating chondrocyte proliferation [21]. Nitric oxide balances osteoblast and adipocyte lineage differentiation by the JNK pathway [22]. The impact of the MAPK pathway during odonto/osteogenic differentiation has drawn a lot of attention in many researchers. ERK, P38, and JNK pathways have been shown to be involved in the odonto/osteogenic differentiation of periodontal ligament stem cells (PDLSCs) when stimulated with mineral trioxide aggregate (MTA) [23]. Similarly, it has been found that bone defects can be reversed by exogenous BMP2 through ERK, P38, and JNK pathways [24]. Therefore, we would like to know whether the MAPK pathway might be involved in the differentiation of SCAPs when stimulated with PTH.

Collectively, the purpose of this study was to uncover the mechanism of PTH action in human SCAPs with respect to proliferation, differentiation, and the MAPK pathway. By understanding the molecular regulatory role of PTH in the committed differentiation of SCAPs, further application of PTH in tooth and bone tissue regeneration could be realizable.

## 2. Materials and Methods

### 2.1. Isolation of SCAPs and Culture

The apical papilla was acquired from impacted third molars with no caries and periodontic diseases at the Oral and Maxillofacial Surgery Department of Jiangsu Provincial Stomatological Hospital with the approval of the Ethical Committee of the Stomatological School of Nanjing Medical University. The apical papilla was gently isolated from surfaces of the developing roots with a pair of tweezers, disposed into 1 mm^3^, and then digested in medium containing 3 mg/mL type I collagenase (Gibco, Life Technologies) and 4 mg/mL trypsin (Gibco, Life Technologies) for 1 hour at 37°C. Then, isolated cells were incubated in alpha minimum essential medium (*α*-MEM, Gibco, Life Technologies) supplemented with 10% fetal bovine serum (FBS, Gibco, Life Technologies), 100 U/mL penicillin, and 100 g/mL streptomycin. Cells were passaged for 3 days a time. Subculture was performed when reaching their confluence of 80%. SCAPs at 3-5 passages were harvested for this study.

### 2.2. Cell Identification

To determine the origin of SCAPs, the passage 1 SCAPs were rinsed with phosphate-buffered solution (PBS) and collected with trypsin without EDTA. Then, cells were incubated with the following antibodies used: CD29-APC, CD-73PE, CD90-PE, CD105-Cy5.5, CD34-FITC, and CD45-PE (all from BD Biosciences, San Jose, CA, USA), and analyzed by BD FACSCalibur (BD Biosciences, San Jose, CA).

### 2.3. Preparation of PTH-Conditioned Medium

Sterile freeze-dried powder (ApexBio, A1129) of recombinant human PTH1-34 (rhPTH1-34) was reconstituted into solution at a concentration of 1.0 × 10^−6^ mol/L using *α*-MEM and stored at -20°C. The solutions were diluted into different concentrations before use. A 48-hour cycle was carried out according to previous studies [25]. Cells were first treated with PTH for 6 hours and then replaced by media without PTH for the remaining 42 hours. Untreated cells cultured in the complete *α*-MEM served as controls. The treated and untreated culture media were changed each for 48 hours throughout the entire experimental period.

### 2.4. Alkaline Phosphate Activity and Staining

ALP activity was performed with an ALP activity assay kit (Jiancheng, Nanjing, China) as previously described [26]. The bicinchoninic acid (BCA) kit (Beyotime, Shanghai, China) was used to normalize the total protein content. Subsequently, rhPTH(1-34)-treated SCAPs at different concentrations were cultured in 96-well plates at 3000 cells/well. Cells were fixed at day 3 and incubated with the BCIP/NBP reagent (Beyotime, Shanghai, China). ALP staining was then performed following the manufacturer's protocol. According to ALP levels, 10^−8^ mol/L was selected as the ideal inductive medium concentration for the subsequent experiments.

### 2.5. Cell Proliferation Assay

SCAPs (3 × 10^3^ cells/well) were cultured in triplicate in a 96-well plate with different concentrations of PTH. Afterwards, CCK-8 (Dojindo, Kyushu Island, Japan) was added to each well at days 0, 1, 3, 5, 7, 9, and 11. A microplate reader (Titertek, Helsinki, Finland) was taken to measure the optical density at a wavelength of 450 nm.

For the EdU incorporation assay, DNA synthesis was detected with a Click-iT™ EdU imaging kit (Invitrogen) according to the manufacturer's manual. In brief, cells were incubated with EdU and fixed with 4% paraformaldehyde (PFA). After the induction with Triton X-100, cells were treated with 1× Apollo reaction cocktail. For the evaluation of proliferating cells, DNA was stained with 200 *μ*L of Hoechst 33342 (5 *μ*g/mL) for 30 minutes and visualized using a fluorescence microscope.

### 2.6. Flow Cytometry

The control cells and cells intermittently treated with PTH were collected for cell cycle detection by FACScan flow cytometry (BD Biosciences, San Jose, CA) according to the manufacturer's manual. Flow cytometry was also used to study the apoptotic characteristics of cells intermittently treated with PTH according to the instructions of the APC-Annexin V Apoptosis Detection Kit (BioLegend). Three independent experiments were conducted.

### 2.7. Alizarin Red Staining

Alizarin red staining was conducted as previously described [27]. Briefly, cells were cultured with four different media (complete medium, PTH-conditioned medium, MM, MM+PTH-conditioned medium) for 2 weeks. MM, also called mineralization-inducing media, is supplemented with 10 mmol/L *β*-glycerophosphate (Sigma-Aldrich), 50 mg/L ascorbic acid (Sigma-Aldrich), and 10 nmol/L dexamethasone (Sigma-Aldrich). And then, cells were fixed in 4% PFA for 30 minutes and then immersed in 2% alizarin red dye for 10 minutes. The calcified nodules that formed were photographed, mounted, and viewed by the spectrophotometric absorbance at 570 nm under phase-contrast microscopy.

### 2.8. Real-Time Reverse Transcription Polymerase Chain Reaction

Cells were cultured with or without PTH for the time indicated. Total RNA was obtained by using the TRIzol reagent (Invitrogen Corporation, USA). cDNA was synthesized with a PrimeScript RT Master Mix kit (TaKaRa Biotechnology, China). Quantitative PCR was run on an ABI 7300 real-time PCR system (Advanced Biosystems, USA) using the SYBR Green PCR Master Mix reagent (TaKaRa, Japan). The primers used are provided in Table 1. *GAPDH* was used as a quality control to normalize other gene expressions.

### 2.9. Western Blot

Cells pretreated with or without PTH were cultured for 3 days and 7 days, respectively, and then collected. To detect the expression of MAPK pathway-related proteins, cells were intermittently treated for 0, 30, 60, and 90 minutes, respectively. To investigate the influence of inhibitors of MAPK pathways, SCAPs were cultured in serum-free medium for 24 hours and then induced in 10^−8^ mol/L PTH or 10^−8^ mol/L PTH+10 *μ*M inhibitor (SP600125 targeting JNK and SB203580 targeting P38). A cell lysis reagent (Beyotime, China) containing the protease inhibitor phenylmethanesulfonyl (PMSF) was taken to lyse the cells. Then, equal amounts of total protein extracts were separated on a 5%-12% resolving SDS-PAGE gel and transferred onto polyvinylidene (PVDF) membranes. After being blocked in 5% bovine serum albumin (BSA) for 2 hours, the membranes were incubated with primary antibodies to detect osteocalcin (OCN, 1 : 1000, Abcam), osteopontin (OPN, 1 : 1000, Abcam), osterix (OSX, 1 : 1000, Abcam), runt-related transcription factor 2 (RUNX2, 1 : 1000, Abcam), type I collagen (COL-I, 1 : 1000, Abcam), dentin sialophosphoprotein (DSPP, 1 : 1000, Bioworld), ERK (#4695, Cell Signaling Technology, USA), p-ERK (#4370, Cell Signaling Technology, USA), JNK (#9252, Cell Signaling Technology, USA), p-JNK (#9255, Cell Signaling Technology, USA), P38 (#8690, Cell Signaling Technology, USA), and p-P38 (#4511, Cell Signaling Technology, USA) overnight at 4°C. Then, membranes were incubated with an appropriate secondary antibody for 1 hour. The blotted bands were detected by the ImageQuant LAS 4000 system (GE Healthcare, USA).

### 2.10. Immunofluorescence Staining

Immunofluorescence analysis was performed as previously described [28]. Briefly, cells were seeded on sterile glass slides in 12-well plates and intermittently treated with PTH. At the expected time point, cells were fixed and permeabilized with 0.5% Triton X-100 for 10 minutes. After washing, cells were blocked and incubated with primary antibodies including OCN (1 : 100, Abcam), OSX (1 : 100, Abcam), and RUNX2 (1 : 100, Abcam) overnight at 4°C. 4,6-Diamidino-2-phenylindole (DAPI, 1 : 1000, Invitrogen) staining was conducted, and images were detected with a confocal microscope (Olympus, Japan).

### 2.11. Statistical Analysis

All quantitative data were analyzed using the SPSS 21.0 software. The one-way ANOVA test was performed to determine the difference between groups. *P* values less than 0.05 were considered to be statistically significant.

## 3. Results

### 3.1. Characterization of SCAPs and Screening for the Optimal PTH Concentration

**Figure 1:**
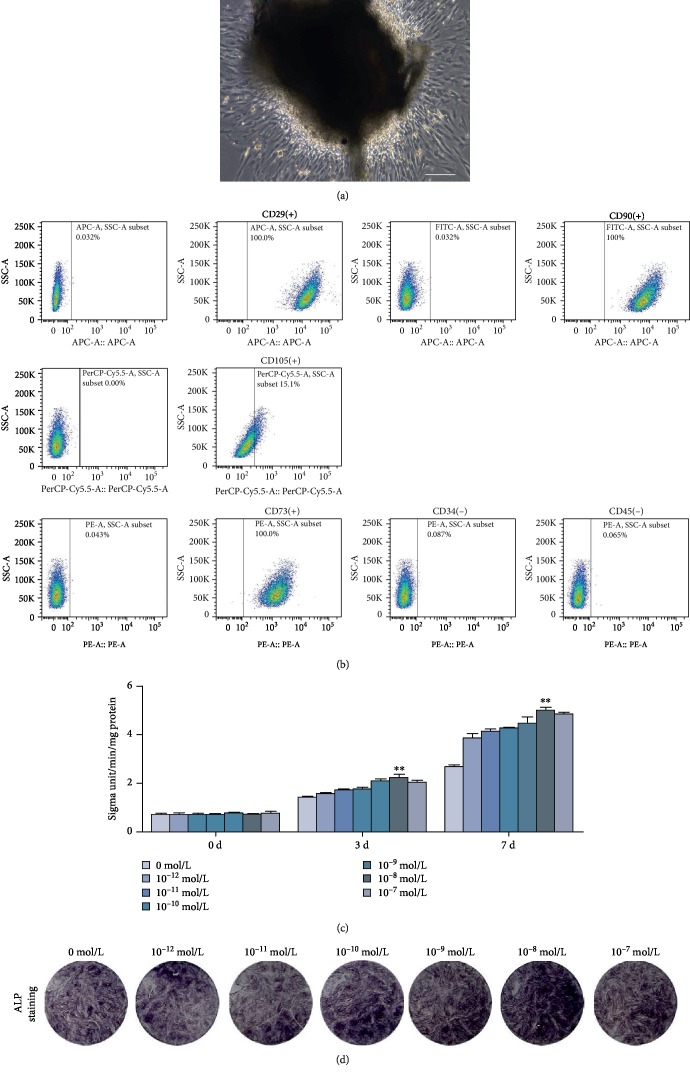
Characterization of SCAPs and selection of the optimal PTH concentration. (a) Morphology of primary SCAPs. Scale bar = 100 *μ*m. (b) Flow cytometry analysis of CD29, CD105, CD90, CD73, CD34, and CD45. (c) ALP activity of SCAPs at days 0, 3, and 7. ^∗∗^*P* < 0.01. (d) ALP staining of SCAPs at day 3.

Primary SCAP morphology was in typical fibroblast- or spindle-like arrangement in the culture flask, and a cell colony in which the cells expand around in a radial pattern was observed (Figure 1(a)). FCM findings revealed that these cells were positive for CD29, CD105, CD90, and CD73 while negative for CD34 and CD45 (Figure 1(b)). To investigate the effects of PTH on the ALP activity, SCAPs were treated with different concentrations of PTH-conditioned media for 3 days. As compared with other groups, the 10^−8^ mol/L PTH group presented the highest ALP activity (Figure 1(c), *P* < 0.01). Images of ALP staining in different concentration groups were scanned with a microscope (Figure 1(d)).

### 3.2. 10^−8^ mol/L PTH Has No Effect on the Proliferation of SCAPs

**Figure 2:**
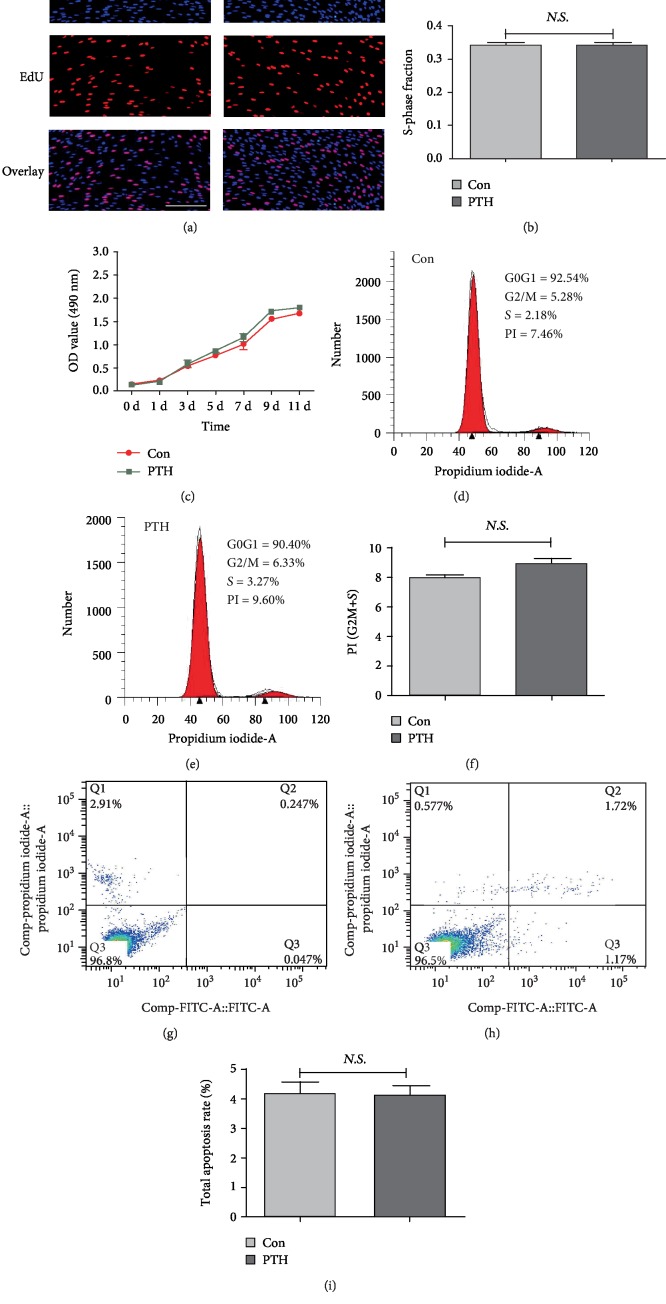
Effects of 10^−8^ mol/L PTH on the proliferation of SCAPs. (a) Representative EdU assay for the control group and PTH group. Scale bar = 50 *μ*m. (b) Synthetic phase fraction of the control group and PTH group. (c) CCK-8 assay for 11 consecutive days (^∗∗^*P* < 0.01). (d) Representative flow cytometry analysis of the cell cycle in the control group. (e) Representative flow cytometry analysis of the cell cycle in the PTH group. (f) Average proliferation index (PI) in the control group and PTH group. Values are means ± SD (*n* = 3). (g) Representative flow cytometry analysis of cell apoptosis in the control group. (h) Representative flow cytometry analysis of cell apoptosis in the PTH group. (i) Total apoptosis rate in the control group and PTH group. Values are means ± SD (*n* = 3).

The EdU retention assay showed no significant difference between the normal media and the 10^−8^ mol/L group (Figures 2(a) and 2(b), *P* > 0.05). In parallel, the CCK-8 assay also revealed that the 10^−8^ mol/L PTH group exerted almost no influence on the cell numbers of SCAPs (Figure 2(c), *P* > 0.05). As indicated by flow cytometry analysis, there was no obvious difference between the control group and the PTH group (Figures 2(d)–2(i), *P* > 0.05). Based on the above data, we therefore applied PTH at the concentration of 10^−8^ mol/L in the following experiments.

### 3.3. PTH Induces the Odonto/Osteogenic Differentiation of SCAPs

**Figure 3:**
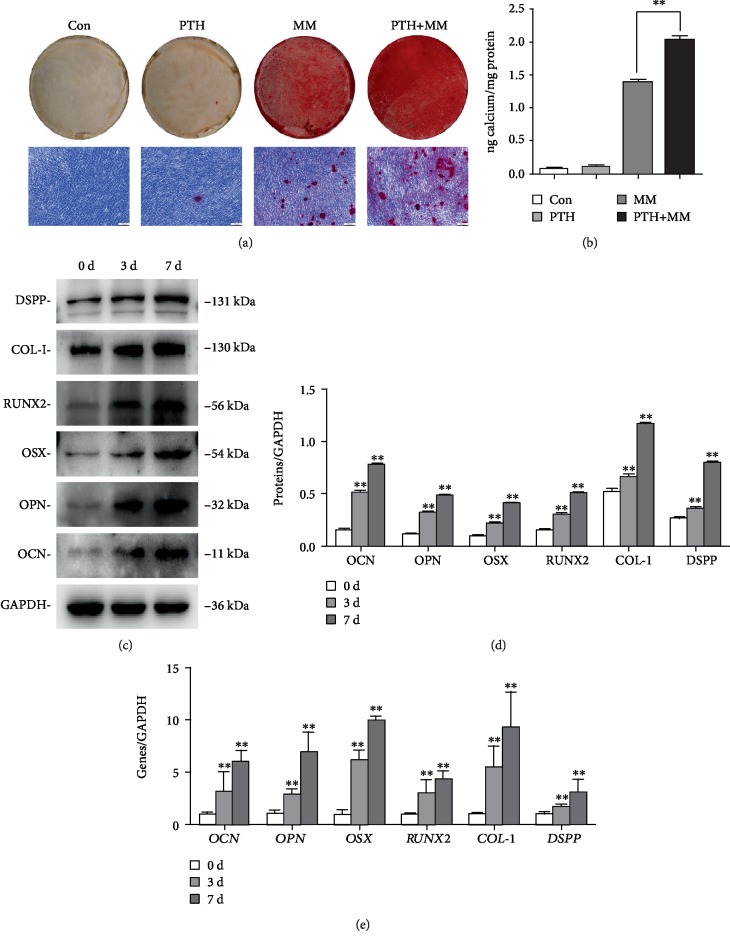
Effects of PTH on odonto/osteogenic differentiation of SCAPs. (a) Representative images of ARS and calcium nodules under the inverted microscope. Scale bar = 100 *μ*m. (b) Quantitative analysis of two groups. Values are means ± SD (*n* = 3). (c) The protein expressions of OCN, OPN, OSX, RUNX2, COL-I, and DSPP in the control group and PTH group at days 0, 3, and 7, respectively. (d) Grayscale analysis of odonto/osteogenic differentiation markers. *n* = 3. ^∗^*P* < 0.05, ^∗∗^*P* < 0.01. (e) The mRNA expressions of *OCN*, *OPN*, *OSX*, *RUNX2*, *COL-I*, and *DSPP* in the two groups. Values are means ± SD (*n* = 3). ^∗∗^2^−ΔΔCt^ > 2, *P* < 0.01; ^∗^1 < 2^−ΔΔCt^ < 2, *P* < 0.01.

The underlying odonto/osteogenic differentiation effect of intermittent treatment of PTH on SCAPs was investigated using alizarin red staining, Western blot, and RT-PCR. Alizarin red staining, indicating matrix mineralization, showed a much higher mineralization reaction in the mineralization induction medium than in the normal medium (Figure 3(a)). Cetylpyridinium chloride (CPC) results also revealed that the osteogenic differentiation potential was stronger in PTH groups than in the control group (Figure 3(b), *P* < 0.01). Additionally, the expressions of odonto/osteogenic-associated markers were analyzed by Western blot and RT-PCR. PTH is inhibiting GAPDH expression where treatments occur over 3 and 7 days. Compared with the control group, PTH markedly upregulate the protein expression of OCN, OPN, OSX, RUNX2, COL-I, and DSPP at day 3 and day 7 (Figures 3(c) and 3(d)). Consistently, the mRNA expressions of *OCN*, *OPN*, *OSX*, *RUNX2*, *COL-I*, and *DSPP* were also markedly increased in a time-dependent manner (Figure 3(e), *P* < 0.01).

### 3.4. PTH Causes the Activation of JNK and P38 MAPK Pathways in SCAPs

**Figure 4:**
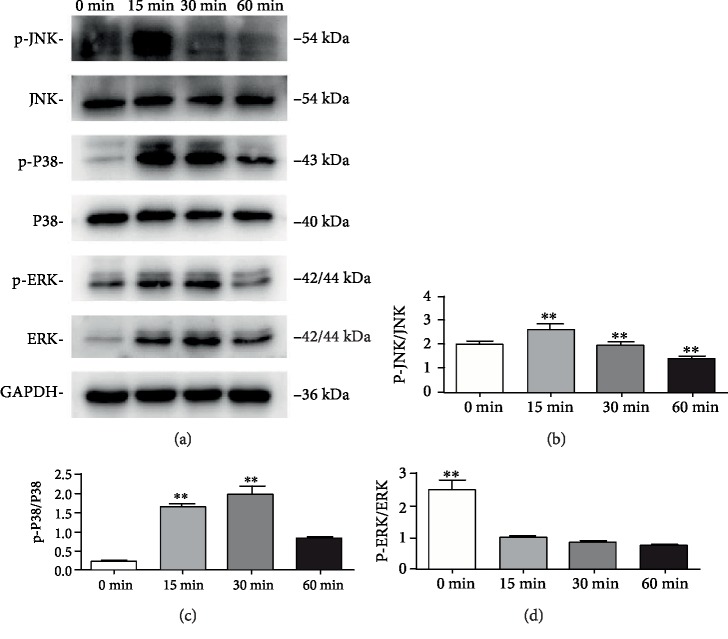
Effects of PTH on the MAPK pathway of SCAPs. (a) The expression levels of p-JNK, JNK, p-P38, P38, p-ERK, and ERK were determined at 0, 15, 30, and 60 minutes, respectively. (b–d) Grayscale analysis of p-JNK/JNK, p-P38/P38, and p-ERK/ERK. GAPDH served as a loading control. *n* = 3. ^∗^*P* < 0.05, ^∗∗^*P* < 0.01.

To investigate whether PTH-induced differentiation was mediated through the MAPK pathway, relative proteins including JNK, P38, and ERK were measured using Western blot. PTH induced the phosphorylation of JNK within 15 minutes of treatment and declined subsequently (Figures 4(a) and 4(b), *P* < 0.05). Activation of P38 was detectable at 15 minutes and reached maximum activation at 30 minutes, then reduced at 60 minutes (Figures 4(a) and 4(c), *P* < 0.05). However, ERK responded negatively to the intermittent administration of PTH in a time-dependent manner, which means that ERK is not activated (Figures 4(a) and 4(d), *P* < 0.05). These results suggest that intermittent PTH treatment increases phosphorylation of JNK and P38.

### 3.5. Inhibition of JNK and P38 MAPK Pathways Inhibits the Odonto/Osteogenic Differentiation of PTH-Treated SCAPs

**Figure 5:**
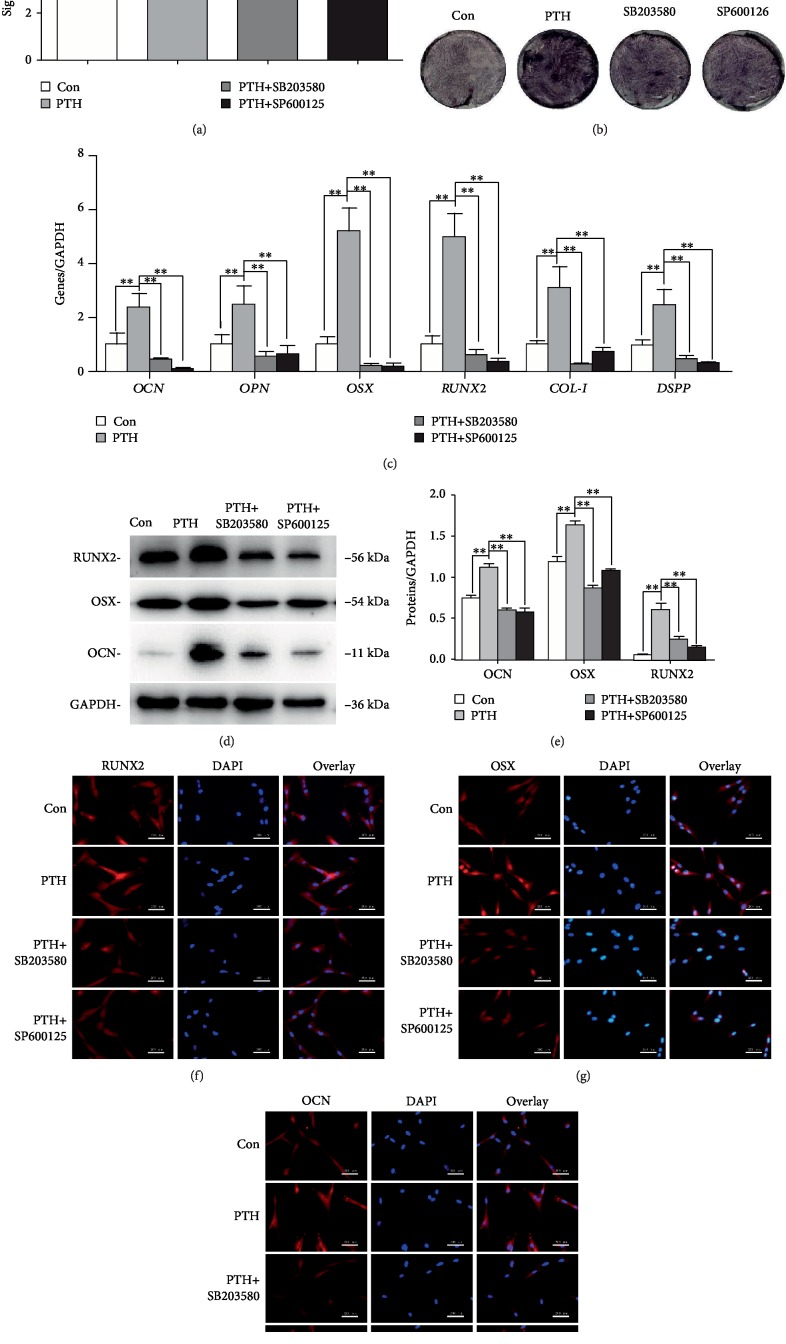
Effects of cotreatment of PTH and MAPK inhibitors on the odonto/osteogenic differentiation of SCAPs. (a, b) ALP activities and ALP staining in the control group, PTH-treated group, and PTH+SB203580 and PTH+SP600125 groups after 3-day culture. Values are means ± SD (*n* = 3). (c) The gene expressions of *OCN*, *OPN*, *OSX*, *RUNX2*, *COL-I*, and *DSPP* at day 7 were determined by RT-PCR. Values are means ± SD (*n* = 3). (d) The protein expressions of OCN, OSX, and RUNX2 in different groups at day 7. (e) Grayscale analysis of the odonto/osteogenic proteins. ^∗^*P* < 0.05, ^∗∗^*P* < 0.01. (f–h) Immunofluorescence staining of OCN, OSX, and RUNX2 in PTH-treated SCAPs and MAPK inhibitors, respectively. Scale bar = 200 *μ*m.

To further confirm that JNK and P38 MAPK pathways mediate the odonto/osteogenic differentiation effects of intermittent PTH on SCAPs, the specific inhibitors SB203580 and SP600125 were used. After 3-day culture, ALP activity significantly increased in the PTH group. However, increased ALP activity was attenuated by concurrent treatment with SB203580 or SP600125 (Figures 5(a) and 5(b)). Western blot and RT-PCR were performed to detect the expression of differentiation markers. The expression of mRNAs was enhanced when treated with PTH whereas it was decreased significantly when cotreated with their respective inhibitors (Figure 5(c)). The above results were further confirmed by Western blot and immunofluorescence analysis (Figures 5(d)–5(h)).

## 4. Discussion

During tooth root development, various growth factors reportedly play vital physiological roles in the dentine formation. For example, IGF-1 can enhance the proliferation and osteogenic differentiation of dental pulp stem cells (DPSCs) and even elongate the molar roots [29]. Estrogen deficiency negatively influences the odonto/osteogenesis, which may result in the impaired mineralization and decreased dental regeneration [30]. In addition, PTH, a major mediator of bone remodeling, when administered intermittently to jaw bones, led to enhanced dental implant osseointegration [31].

PTH1-34 is one of the clinically approved treatment regimens by the US Food and Drug Administration (FDA) for osteoporosis [32]. Because of the advantage of its bone formation capacity, it is bound to be used in broader areas. Moreover, dentine and bone are similar connective tissues that closely resemble each other in composition and developed mechanism [33]. Hence, the purpose of this study was to investigate the effects of intermittent treatment with PTH1-34 on odonto/osteogenic differentiation of SCAPs.

Reports have found that intermittent low-dose PTH enhanced osteogenesis while the higher concentration of PTH might reduce bone formation [34]. ALP is considered to be an early marker for cell-mediated mineralization. Increased ALP activity values at early culture stages indicated that dental mesenchymal cells are highly differentiated [35]. According to our ALP activity results, 10^−8^ mol/L was the optimal intermittent inducing concentration of PTH on SCAPs. Besides, cell growth rates were assessed to detect the proliferation effect of 10^−8^ mol/L PTH on SCAPs. Our observations showed that intermittent administration of PTH had no effect on the proliferation of SCAPs as indicated by CCK-8, EdU, and FCM.

To determine the odonto/osteogenic differentiation capacity of PTH, alizarin red staining, RT-PCR, and Western blot were performed. As calcium nodule production is a marker of mineralization, the increased formation of mineralized nodules indirectly indicated the critical role of PTH in the mineralization and differentiation of SCAPs [36]. RT-PCR and Western blot have shown induction of odonto/osteogenic differentiation indicators (OCN, OPN, OSX, RUNX2, COL-I, and DSPP) after PTH treatment. OCN was detected throughout the length of the odontoblast processes and always appeared in the enamel when the maturation age is reached [37]. OPN, a single-chain polypeptide, is highly concentrated in the mineral surface [38]. OSX, a zinc-finger-containing transcription factor, is expressed in dental mesenchymal cells. Multiple studies have found that cortical bone formation is abolished and osteoblast marker genes are reduced in OSX-null mice [39]. RUNX2 acts as an upstream gene of OSX. RUNX2 directly regulates the expression of multiple odontoblast genes [40]. COL-I is broadly distributed in bone, cartilage, and dentine [41]. DSPP, a major odontoblastic cell marker, plays an essential role in dentinogenesis and is formed in the dentine extracellular matrix [42]. The significant upregulation of mineralization markers indicated the odonto/osteogenic inductive effects of PTH in SCAPs.

Multiple signaling pathways are involved in the differentiation process of SCAPs, including MAPK. The MAPK pathway can transduce extracellular stimuli into the nucleus and control gene expression to promote differentiation. It is reported that P38 is activated by bone morphogenetic protein 2 (BMP2) during odontoblast stimulation in tertiary dentinogenesis [43]. Another study indicated that the inhibition of P38 notably antagonized lipopolysaccharide- (LPS-) induced dentine matrix-associated markers [44]. Furthermore, the JNK subclass of MAPKs may be elevated basally in response to cytokines. Recent studies showed that JNK plays a role in calcium hydroxide-induced mineralization in DPSCs [45]. Biochemical studies confirmed that JNK may trigger Dpp expression by activation of the AP-1 complex [46]. The role of ERK activation seems to be controversial. Work from several laboratories showed that ERK activation was essential for osteogenesis whereas other reports have detected that ERK activation inhibited the regulation of osteoblast functions [47, 48]. Studies showed that downregulation of p-ERK in BMSCs can induce osteogenesis when treated with BMP2 and PTH [49]. Our data showed that p-ERK was downregulated by intermittent PTH administration, which is consistent with the hypothesis that ERK dephosphorylation induces bone formation. Finally, this study indicated that the presence of intermittent PTH induced the phosphorylation of P38 and JNK while the suppression of P38 and JNK attenuated PTH-induced phosphorylation and nuclear translocation of MAPK. Taken together, the above results suggest that PTH induces the activation of JNK and P38 MAPK pathways in SCAPs.

Since SCAP mineralization accompanies the intermittent stimulation of PTH, we assumed that PTH might be involved in SCAP differentiation. In conclusion, our findings illustrate the role of PTH in the mineralization of SCAPs through the MAPK pathway, providing an attractive approach to promote tooth and bone tissue regeneration research by regulating cell biological behavior. However, we only conducted the *in vitro* test in this study. Further studies in animal models and clinical endodontic treatment are required to clarify the exact role of PTH in regenerative medicine.

## Figures and Tables

**Table 1:** Sense and antisense primers for real-time reverse transcription polymerase chain reaction.

Genes	Primers	Sequences (5′-3′)
*DSPP*	Forward	ATATTGAGGGCTGGAATGGGGA
	Reverse	TTTGTGGCTCCAGCATTGTCA

*COL-I*	Forward	CCCTTTCTGCTCCTTTCT
	Reverse	TGTTTCCTGTGTCTTCTGG

*RUNX2*	Forward	TCTTAGAACAAATTCTGCCCTTT
	Reverse	TGCTTTGGTCTTGAAATCACA

*OSX*	Forward	CCTCCTCAGCTCACCTTCTC
	Reverse	GTTGGGAGCCCAAATAGAAA

*OPN*	Forward	CCTGACTATCAATCACATCGGAAT
	Reverse	TGACCAGAGTGCTGAAACCCA

*OCN*	Forward	AGCAAAGGTGCAGCCTTTGT
	Reverse	GCGCCTGGGTCTCTTCACT

*GAPDH*	Forward	GAAGGTGAAGGTCGGAGTC
	Reverse	GAGATGGTGATGGGATTTC

## Data Availability

The data used to support the findings of this study are available from the corresponding authors upon request. The readers can contact Professor Yu via email to obtain data.
